# Supplementary material for: Marine ecosystem role in setting up preindustrial and future climate
Source: Nat Commun. 2025 Mar 5;16:2206. doi: 10.1038/s41467-025-57371-y (PMC11883021; doi:10.1038/s41467-025-57371-y)
Supplement: Supplementary file 1 — Supplementary Information [file 41467_2025_57371_MOESM1_ESM.pdf]

Supplementary material for "Marine ecosystem role in setting up preindustrial and future climate"

*Jerry Tjiputra[a], Damien Couespel[a], Richard Sanders[a]

[a]NORCE Norwegian Research Centre AS, Bjerknes Centre for Climate Research, Bergen, Norway
Corresponding author: jetj@norceresearch.no

Content of this file

Supplementary Tables 1 and 2

Supplementary Figures 1 to 11

Table 1. Long-term drift of global mean climate state and carbon budgets in *REF* and *Abiotic* simulations. Values are estimated from 250 years of preindustrial control simulations (i.e., the difference between mean values from the the last and the first 50 years divided by 200 years).

Variables	Reference	Abiotic	Units
Surface air temperature	0.00	0.00	°C yr^{-1}
Atmospheric CO_2	-0.01	0.03	ppm yr^{-1}
Sea surface temperature	0.00	0.00	°C yr^{-1}
Maximum AMOC[1]	0.00	0.00	Sv yr^{-1}
Dissolved inorganic carbon[2]	-0.14	0.04	Pg C yr^{-1}
Marine carbon sediment	0.87	-0.12	Pg C yr^{-1}
Land vegetation carbon	-0.01	0.04	Pg C yr^{-1}
Land soil and litter carbon	-0.06	-0.03	Pg C yr^{-1}
Arctic sea-ice area	0.00	0.00	10^6 km^2 yr^{-1}
Antarctic sea-ice area	0.00	0.00	10^6 km^2 yr^{-1}

[1] Atlantic Meridional Overturning Circulation at 26°N, [2] In the water column.

Table 2. Global mean projected change in key climate metrics and carbon budgets in *REF* and *Abiotic* simulations. The projected change are difference between 2091-2100 and 1851-1890 periods from the both SSP1-2.6 and SSP2-4.5 future scenarios and Historical simulations.

	Δ (SSP1-2.6)		Δ (SSP2-4.5)		
Variables	Reference	Abiotic	Reference	Abiotic	Units
Surface air temperature	1.15	1.85	2.00	2.78	°C
Atmospheric CO_2	138	245	287	433	ppm
Sea surface temperature	0.79	1.37	1.34	1.97	°C
Ocean temperature	0.20	0.45	0.24	0.50	°C
Maximum AMOC[1]	-7.59	-7.63	-8.06	-8.30	Sv
Dissolved inorganic carbon[2]	261	240	357	305	Pg C
Marine carbon sediment	205	-29	204	-29	Pg C
Sea-to-air CO_2 flux	-0.30	-0.21	-2.08	-1.36	Pg C yr^{-1}
Surface ocean Revelle factor	1.61	1.84	2.22	2.34	-
Land vegetation carbon	91	47	110	37	Pg C
Land soil and litter carbon	58	-11	71	-17	Pg C
Land-to-air CO_2 flux	-1.61	-1.51	-3.08	-2.15	Pg C yr^{-1}
Arctic sea-ice area	-1.46	-2.41	-3.11	-4.49	10^6 km^2
Antarctic sea-ice area	-0.01	-1.27	-0.26	-2.08	10^6 km^2

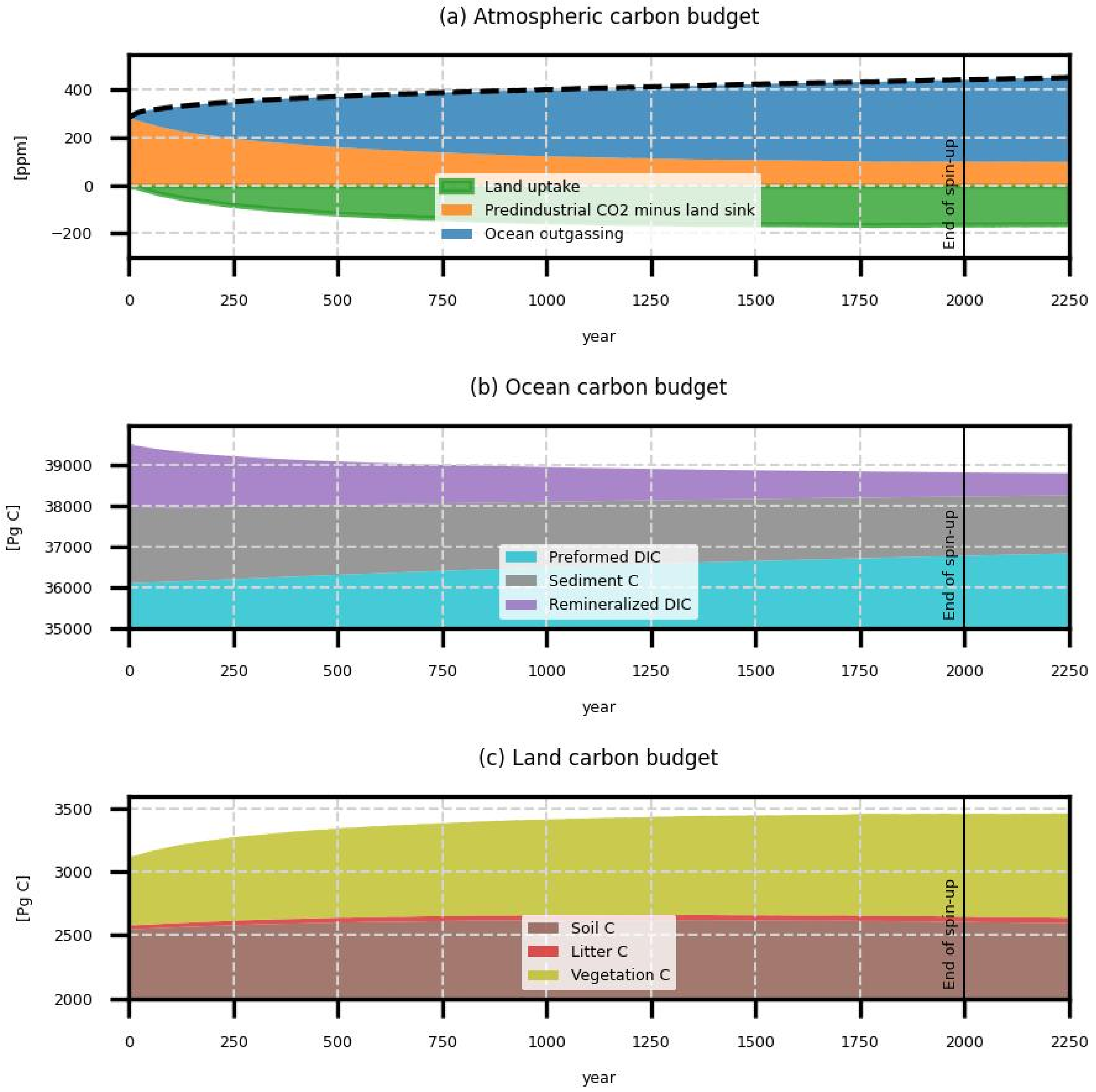

Figure 1. Evolution of CO_2 during equilibration period in the atmosphere, ocean, and land. The evolution of the (a) atmosphere, (b) ocean, and (c) land carbon budget during the 2000 years model spin-up after the ocean biological productivity is deactivated (*Abiotic*). The years 2001-2250 represent the subsequent preindustrial control simulation.

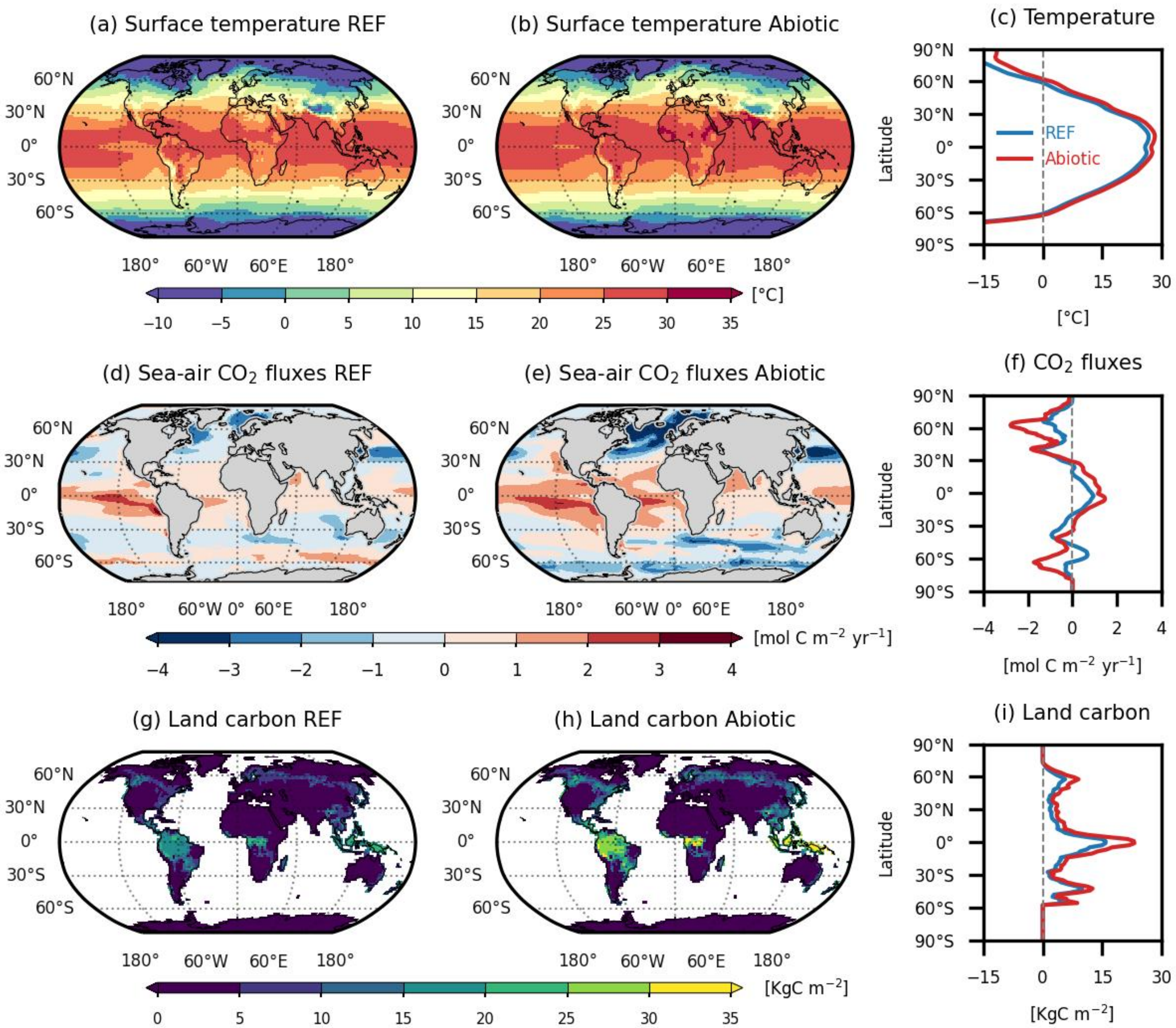

Figure 2. Preindustrial surface temperature and CO_2 fluxes. Annual mean preindustrial distribution of (a,b,c) surface air temperature, (d,e,f) sea-to-air CO_2 fluxes and (g,h,i) land vegetation carbon pool. Shown are values from (a,d,g) *REF* and (b,e,h) *Abiotic* simulations. Panels (c,f,i) depict zonally averaged values. All values are averaged over the first 30 years preindustrial control period (1851-1880).

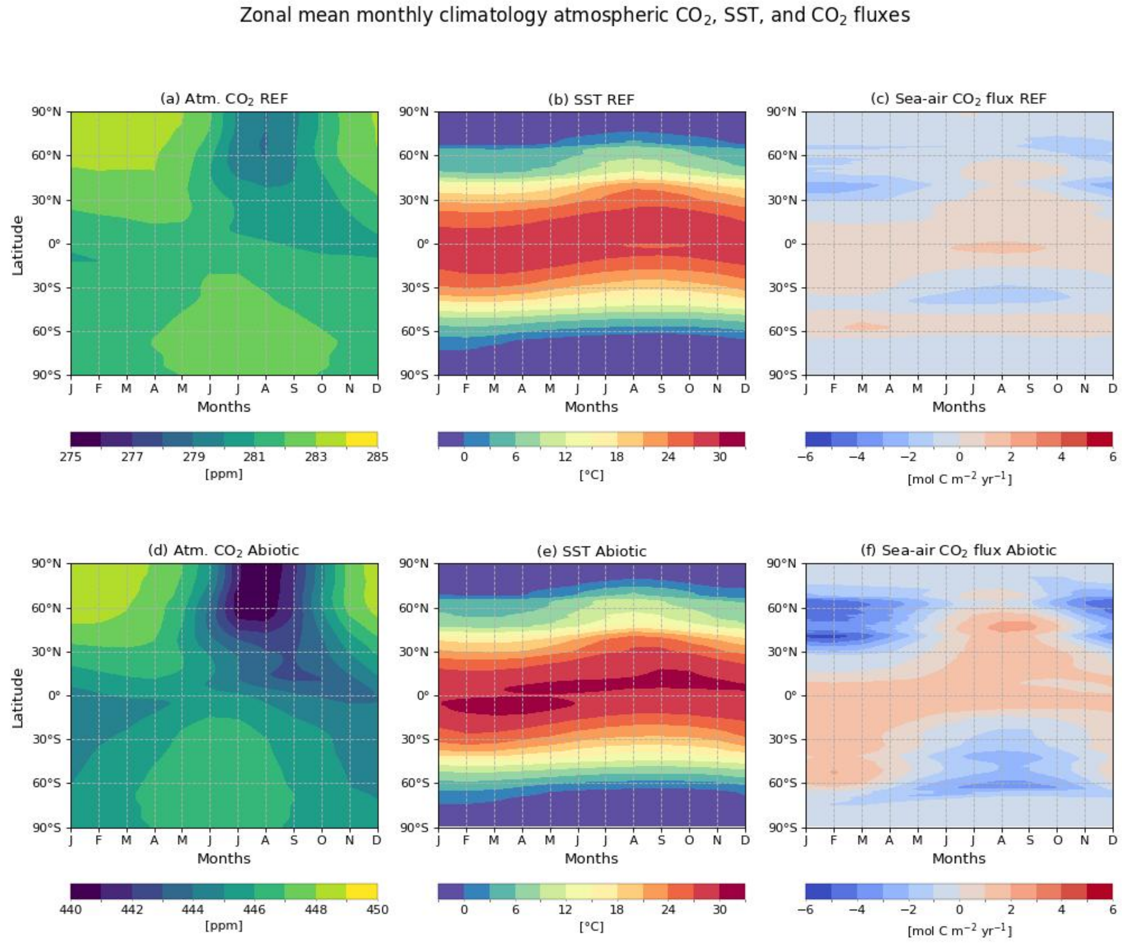

Figure 3. Seasonal variability of $CO2_2$, temperature and CO_2 fluxes. Preindustrial Zonally averaged (a) atmospheric CO_2 concentration, (b) sea surface temperature (SST), and (c) sea-air CO_2 fluxes from the *REF* simulation. Panels (d,e,f) depict respective values from *Abiotic* simulation. Shown are the first 30-year average values from the preindustrial control simulations.

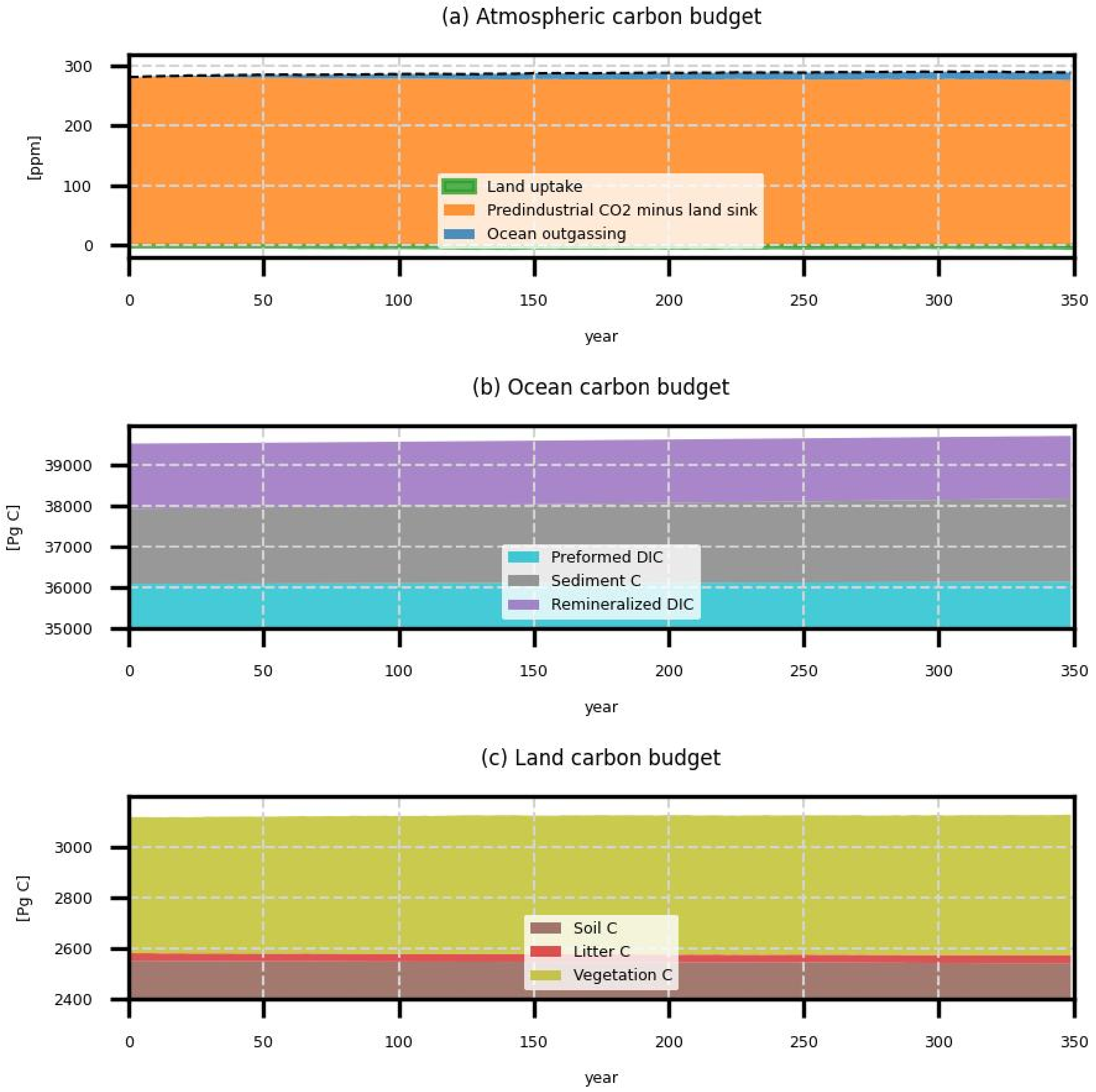

Figure 4. Evolution of CO_2 during equilibration period in the atmosphere, ocean, and land with 90% biology. The evolution of preindustrial (a) atmosphere, (b) ocean, and (c) land carbon budget during the 350 years model spin-up after the ocean biological productivity is reduced by 10%, i.e., the prognostic primary production is multiplied by a factor 0.9.

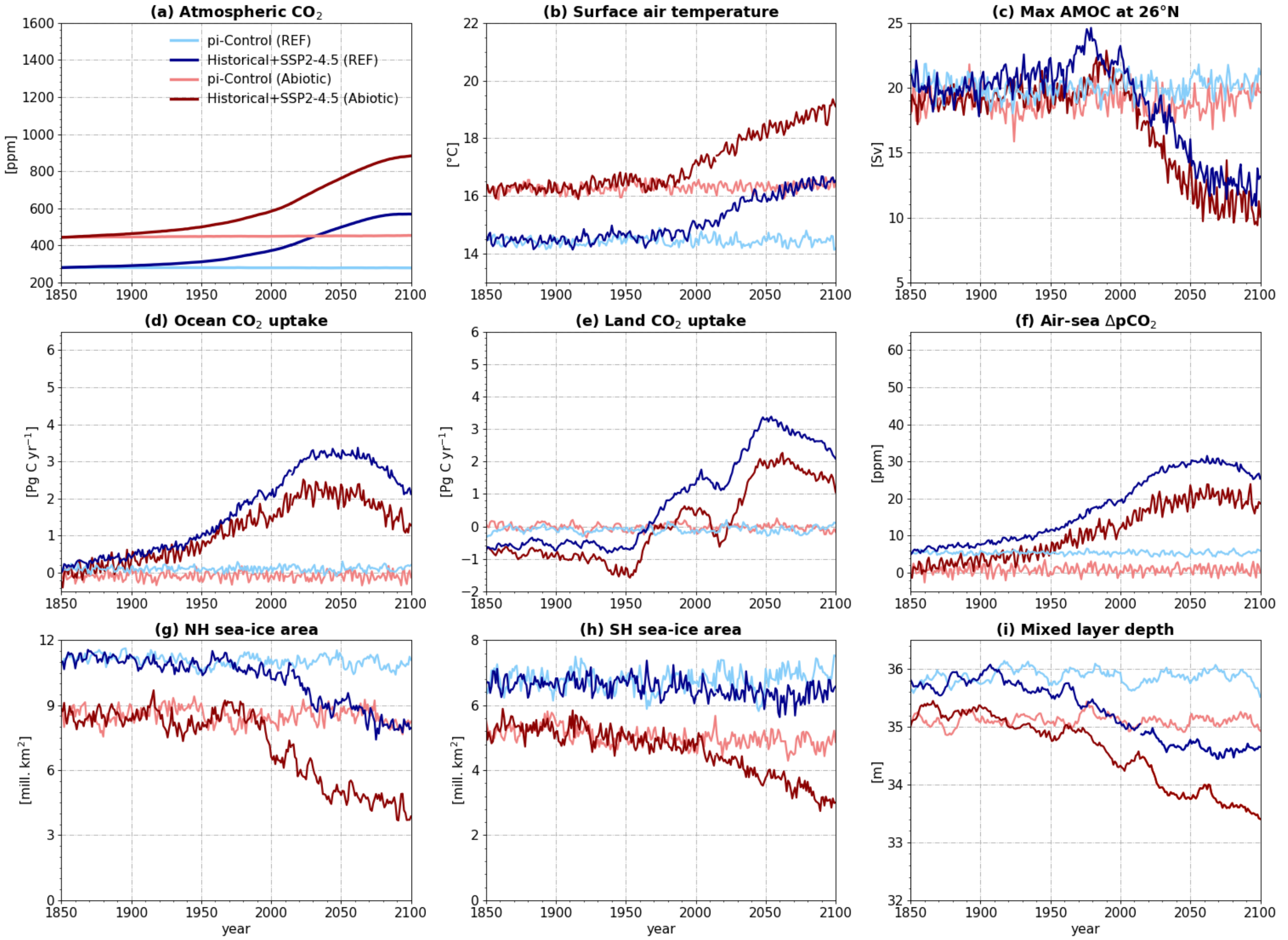

Figure 5. Historical and future projections of global climate and carbon cycle states. Time-series of global annual mean (a) atmospheric CO_2 concentrations, (b) surface air temperatures, (c), Atlantic Meridional Overturning Circulation strengths, (d) ocean CO_2 uptakes, (e) 10-yr running mean of land CO_2 uptakes, (f) air-sea ΔpCO_2, (g) northern hemisphere sea-ice area, (h) southern hemisphere sea-ice area, and (i) ocean mixed layer depths for *REF* (blue-lines) and *Abiotic* (red-lines) NorESM2-LM (Norwegian Earth System Model) simulations under pre-industrial control, historical, and SSP2-4.5 scenarios.

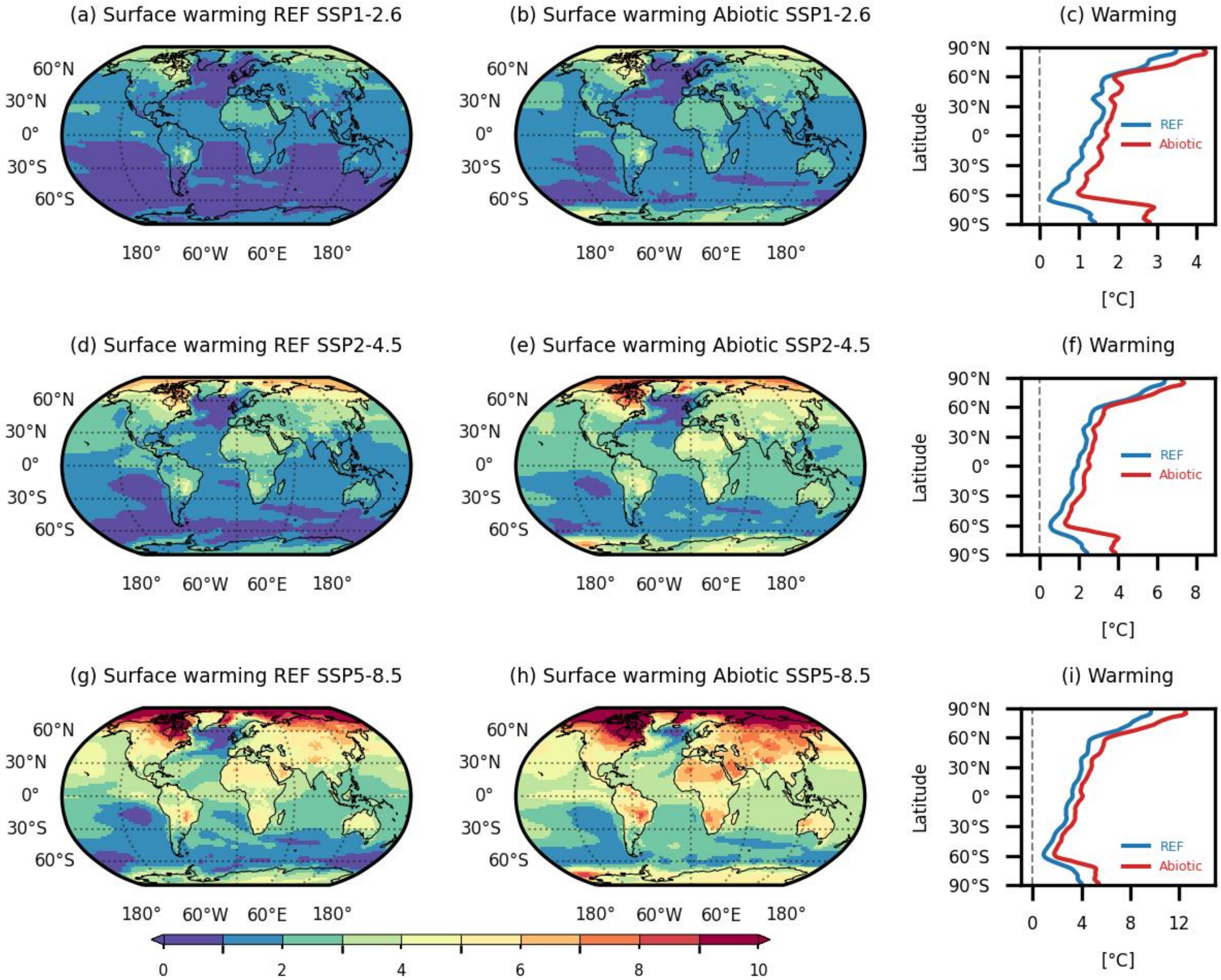

Figure 6. Projections of surface warming. Maps of projected surface air temperature change at the end of the 21st century (2071-2100 minus 1851-1880) under the (a,b,c) SSP1-2.6, (d,e,f) SSP2-4.5 and (g,h,i) SSP5-8.5 scenarios for the *REF* and *Abiotic* simulations. Panels (c,f,i) depict the zonally average values.

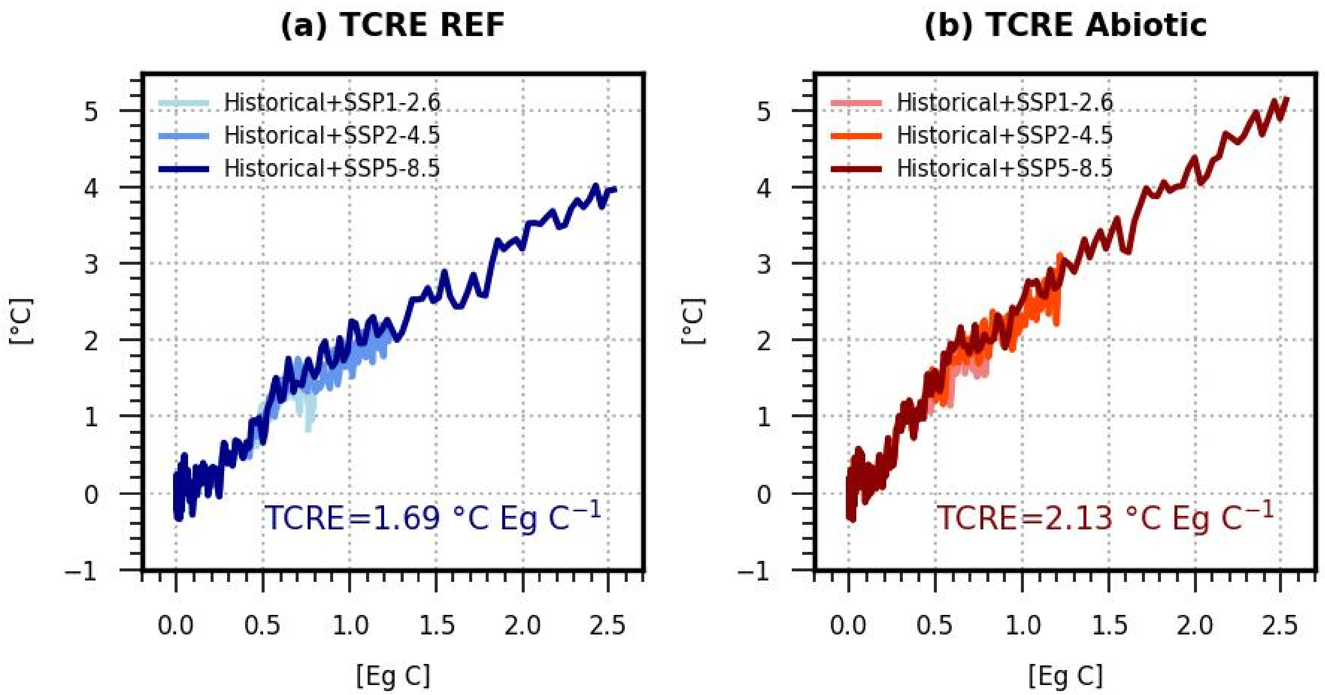

Figure 7. Transient climate response to cumulative CO_2 emissions (TCRE). TCRE from (a) *REF* and (b) *Abiotic* simulations over the historical, SSP1-2.6, SSP2-4.5, and SSP5-8.5 scenarios. Shown are global mean surface air temperature warming relative to the preindustrial period (y-axes) vs cumulative anthropogenic CO_2 emissions since the preindustrial. The TCRE values are estimated by applying linear regression to the historical and SSP5-8.5 experiments.

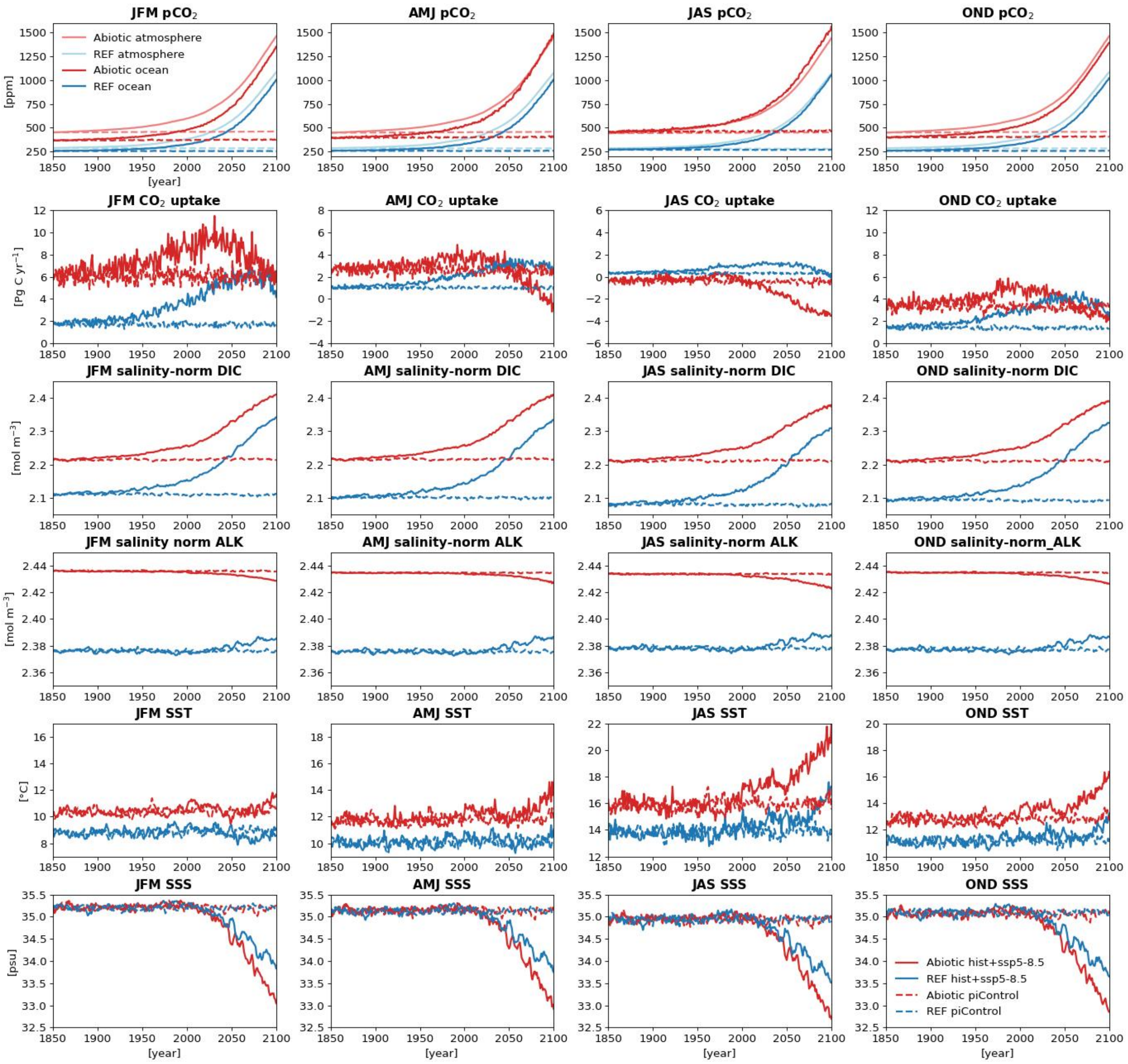

Figure 8. Changes in ocean carbon cycle and hydrography across seasons. Transient evolution (1850-2100) of North Atlantic (1st-row) atmospheric (dark colors) and surface ocean (light colors) pCO_2, (2nd-row) oceanic CO_2 uptake, (3rd-row) salinity-normalized surface dissolved inorganic carbon (DIC), (4th-row) salinity normalized surface alkalinity, (5th-row) sea surface temperature (SST), and (6th-row) sea surface salinity (SSS). Shown are values averaged for each season for the historical and SSP5-8.5 experiments: (1st-column) January-February-March (JFM), (2nd-column) April-May-June (AMJ), (3rd-column) July-August-September (JAS), and (4th-column) October-November-December (OND). The North Atlantic domain is defined in Fig. 3c (green-dashed outline). Blue and red colors depict values from *REF* and *Abiotic* simulations, Dashed and solid lines represent preindustrial control and historical+SSP5-8.5 projections, respectively.

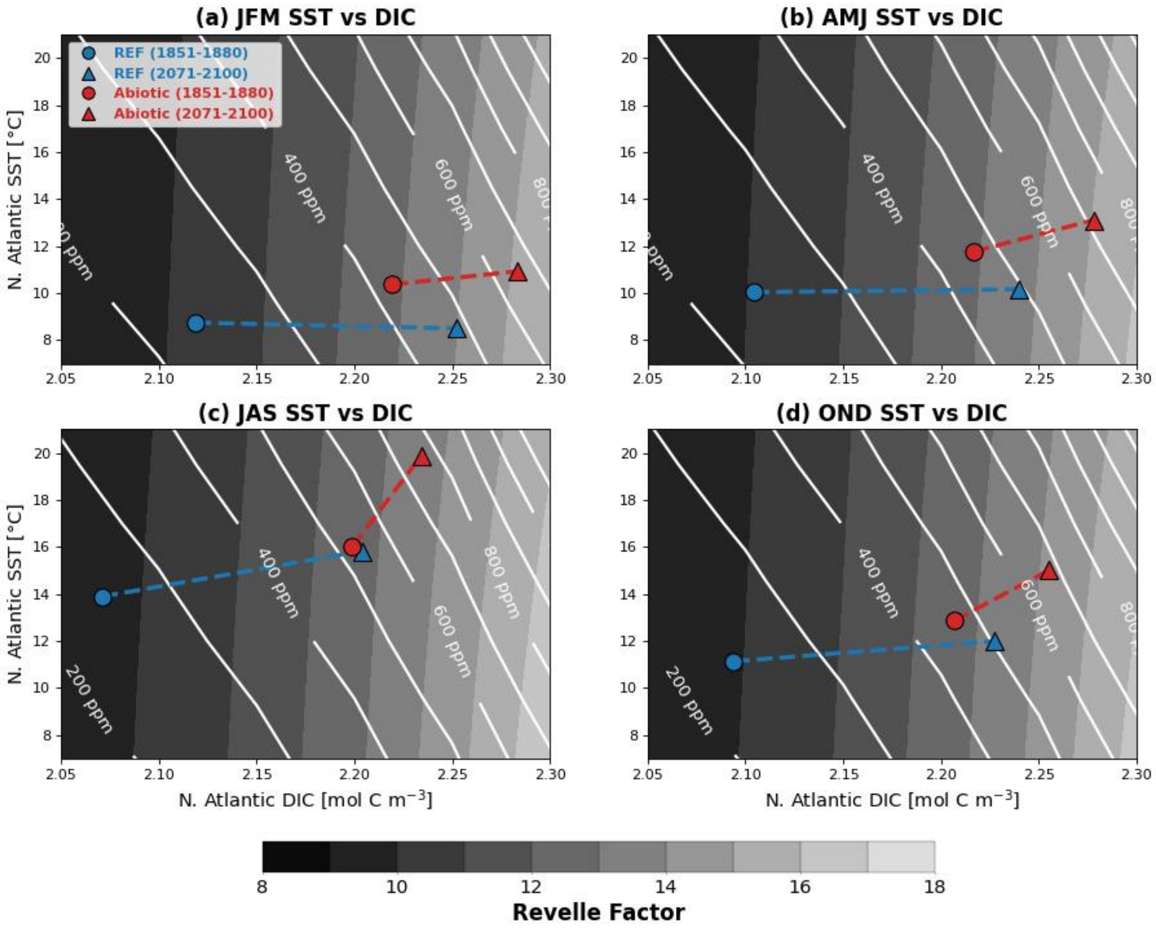

Figure 9. Changes in Revelle factor. Simulated changes in sea surface temperature and dissolved inorganic carbon concentration in the subpolar North Atlantic from the beginning of the historical (1851-1880) to the end of the 21st century (2071-2100) under SSP5-8.5 scenario for (a) Winter - JFM (January-February-March), (b) Spring - AMJ (April-May-June), (c) Summer - JAS (July-August-September), and (d) Fall - OND (October-November-December) seasons. The overlay surface ocean pCO_2 (white contours) and Revelle Factor (gray shadings) are calculated using mean salinity and alkalinity from the early historical period (1851-1880). The North Atlantic domain is defined in Fig. 3c (green-dashed outline).

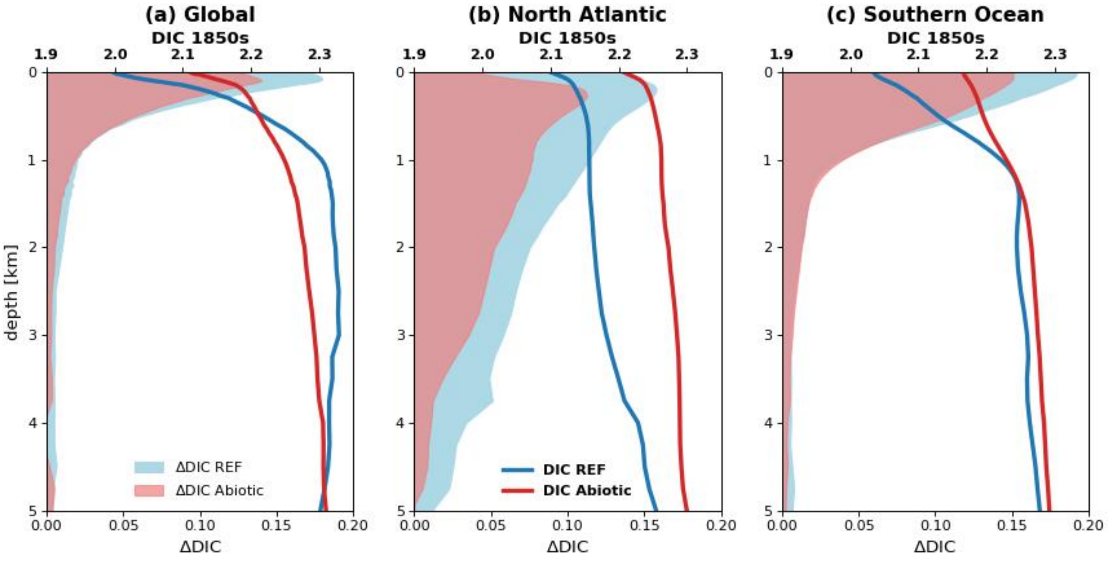

Figure 10. Vertical profiles of dissolved inorganic carbon (DIC). Vertical DIC profiles averaged for the (a) global ocean, (b) North Atlantic, as outlined in Fig. 2c, and (c) Southern Ocean, between 30°S and 55°S averaged over the start of historical period (1851-1860, top x-axes) for the (blue lines) *REF* and (red lines) *Abiotic* experiments. Color shadings (bottom x-axes) depict increases in vertical DIC concentrations by the end of the 21st century under the SSP5-8.5 scenario (2091-2100 minus 1851-1860). Units are in [mol C m^{-3}].

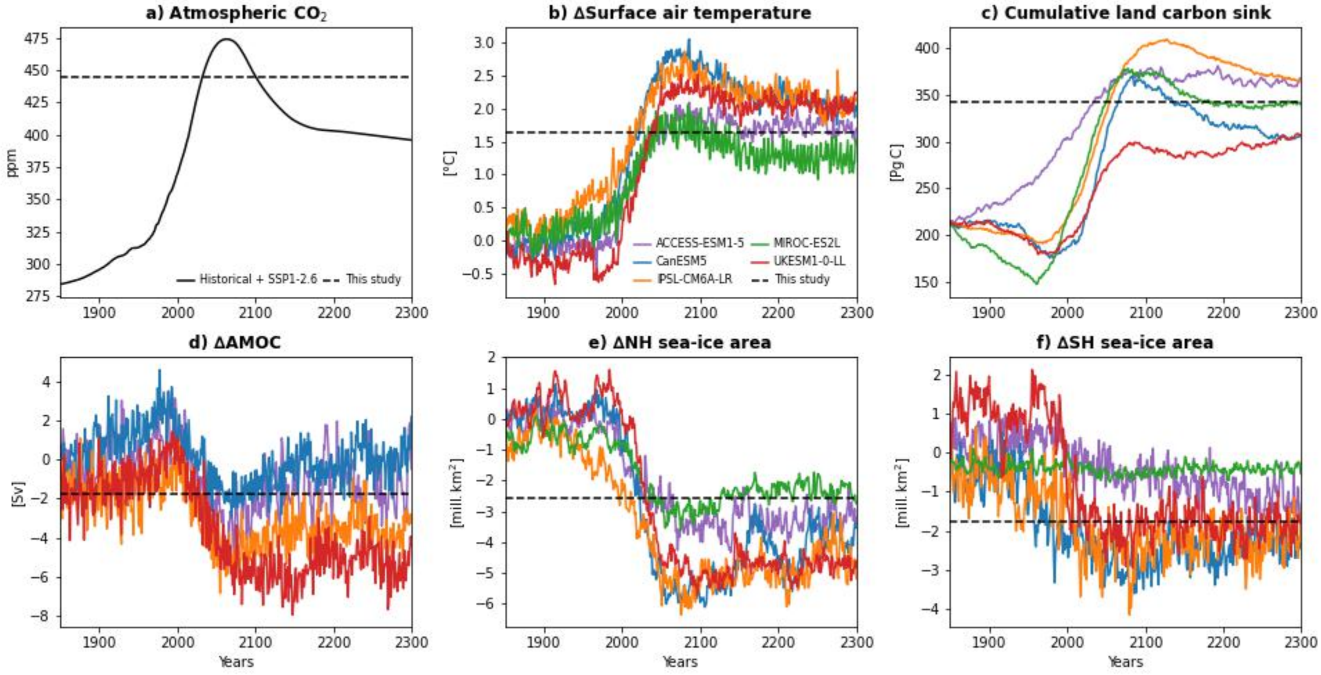

Figure 11. Time-series of climate change and land sink in CMIP6 (Coupled Model Intercomparison Project phase 6). The evolution of global mean (a) atmospheric CO_2, (b) change in surface air temperature, (c) cumulative change in land carbon budget, (d) change in Atlantic Meridional Overturning Circulation strength, (e) change in Northern Hemisphere and (f) Southern Hemisphere sea-ice area as simulated in five CMIP6 Earth System Models under the historical and extended SSP1-2.6 scenarios. For (c), we have added 215 Pg C, which reflects estimate of land-use change carbon loss from 1850-2150 period.
